# Supplementary material for: Mass action model of solution activity via speciation by solvation and ion pairing equilibria
Source: Commun Chem. 2021 Nov 26;4:163. doi: 10.1038/s42004-021-00599-8 (PMC9814931; doi:10.1038/s42004-021-00599-8)
Supplement: Supplementary file 1 — Supplementary Information [file 42004_2021_599_MOESM1_ESM.pdf]

Supplementary Methods for

Mass Action Model of Solution Activity via Speciation by Solvation and Ion Pairing Equilibria

Aaron D. Wilson*, Hyeonseok Lee, Caleb Stetson
Idaho National Laboratory, P.O. Box 1625 MS 2208, Idaho Falls, ID 83415-2208, USA
*Corresponding author: aaron.wilson@inl.gov

Table S1. *Calculated $\mathrm{K_{ha}}$ and $\mathrm{K_{id}}$ using VLE Data at 298.15K.*

K_{ha}* (K_{id}) {lit K_{id}}†	H^+	Li^+	Na^+	K^+	Rb^+	Cs^+	NH_4^+
NO_3^-	2.15 (0.41)	3.19 (0.35)	1.34 (0.025)	0.0# (0.0058)	0.0 (0.0031)	0.0# (0.0080)	0.23 (0.022)
F^-			4.16# (0.012)	3.53 (0.051)	3.50# (0.045)		
Cl^-	3.80 ($10^{6.3}$)**	$4.78x_A^{2.41}$ 0.10** {0.041}	3.67 (0.033) {0.011}	2.89 (0.025) {0.012}	2.73 (0.024) {0.014}	2.45 (0.022) {0.018}	2.37 (0.036)
Br^-	4.09 ($10^{8.7}$)**	$5.08x_A^{2.45}$ 0.12**	4.16 (0.033)	2.85 (0.031)	2.51 (0.024)	2.50 (0.017)	3.34# (0.022)
I^-	4.48# ($10^{9.4}$)**	$5.32x_A^{2.40\#}$ 0.15**	4.60# (0.039) {0.053}	3.00 (0.037) {0.025}	2.59 (0.023)	2.06# (0.017) {0.014}	
OH^-		2.53 (0.020)	3.83 (0.097)	3.82 (0.52)		3.39# (0.043)	

*$K_{ha}x_A{}^2$ unless otherwise indicated. **Values manually fixed to order of magnitude to literature and periodic trend. #Data fitting limited to 3.5 molal and may be non-convergent. †{lit $\boldsymbol{K_{id}}$} Dissociation constants obtained from electrochemical experiments. [Gujt, J., Bešter-Rogač, M. & Hribar-Lee, B. An investigation of ion-pairing of alkali metal halides in aqueous solutions using the electrical conductivity and the Monte Carlo computer simulation methods. *J. Mol. Liq.* **190**, 34–41 (2014)].

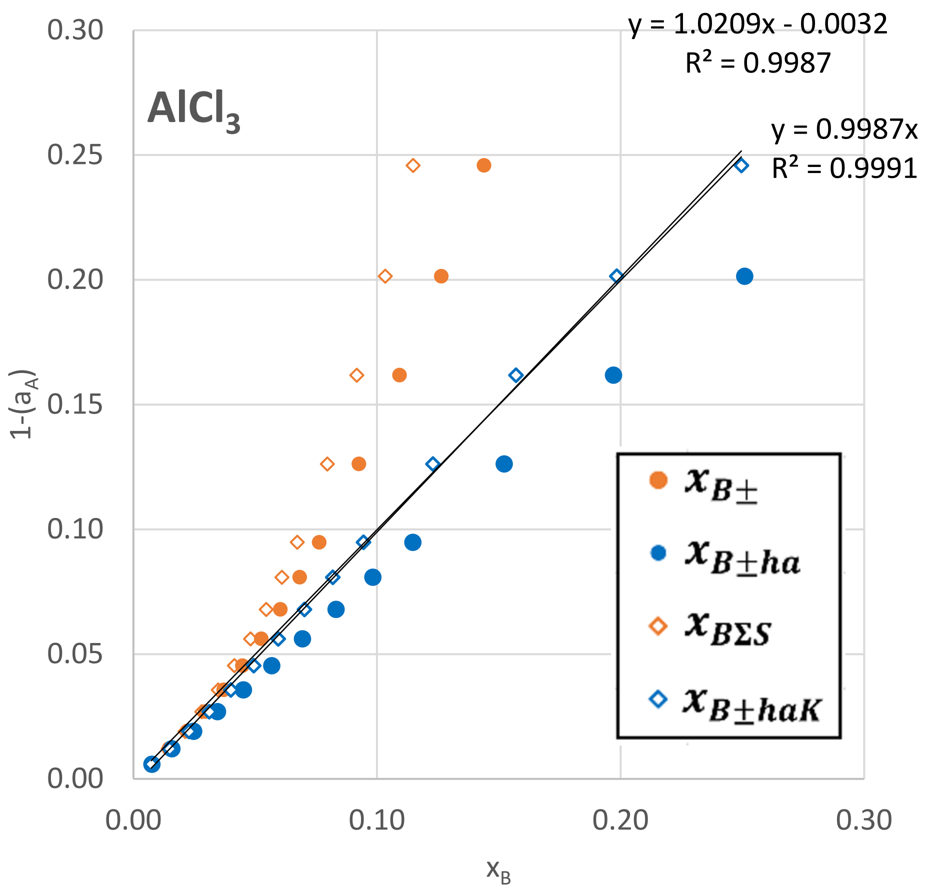

Figure S1. $AlCl_3$ activity data (Robinson, R. A. & Stokes, R. H. *Electrolyte Solutions: Second Revised Edition*. (Dover Publications, Incorporated, 2012)) fit to $K_{id} = 0.027$, $K_{ha} = 7.08$.

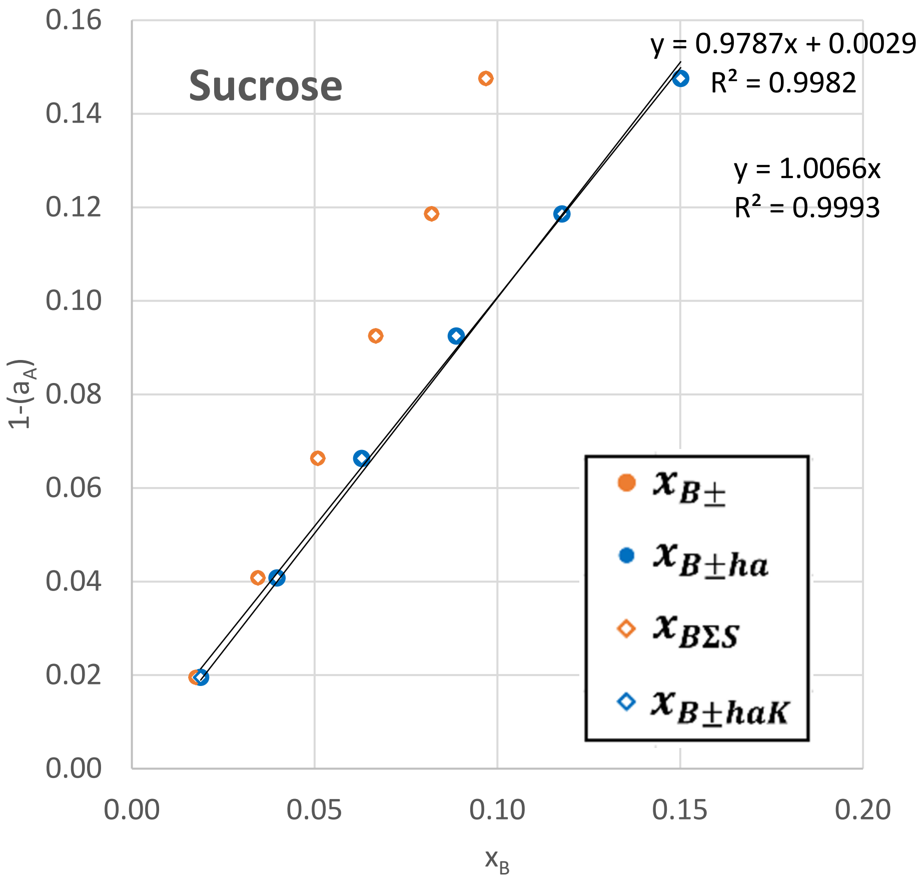

Figure S2. Sucrose activity data (Robinson, R. A. & Stokes, R. H. *Electrolyte Solutions: Second Revised Edition*. (Dover Publications, Incorporated, 2012)) fit to $K_{ha} = 3.824$.

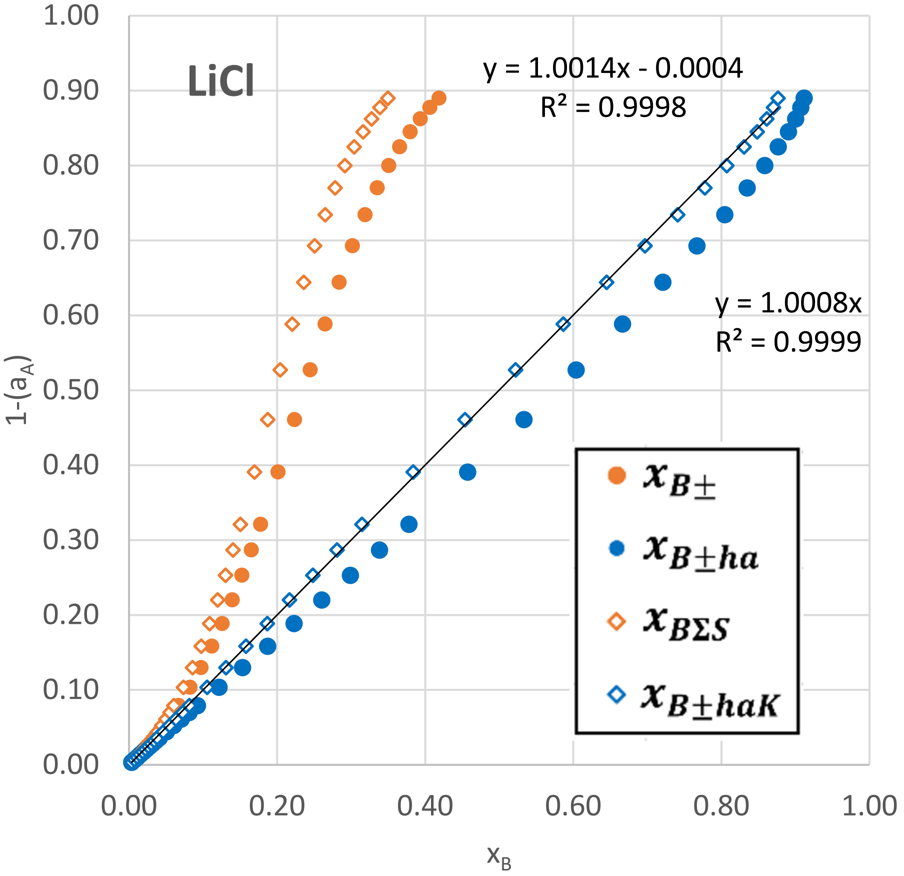

Figure S3. LiCl activity data (Robinson, R. A. & Stokes, R. H. *Electrolyte Solutions: Second Revised Edition*. (Dover Publications, Incorporated, 2012)) fit to $K_{id} = 0.1(\text{fixed})$, $K_{ha} = 4.78$, *m* =2.41.

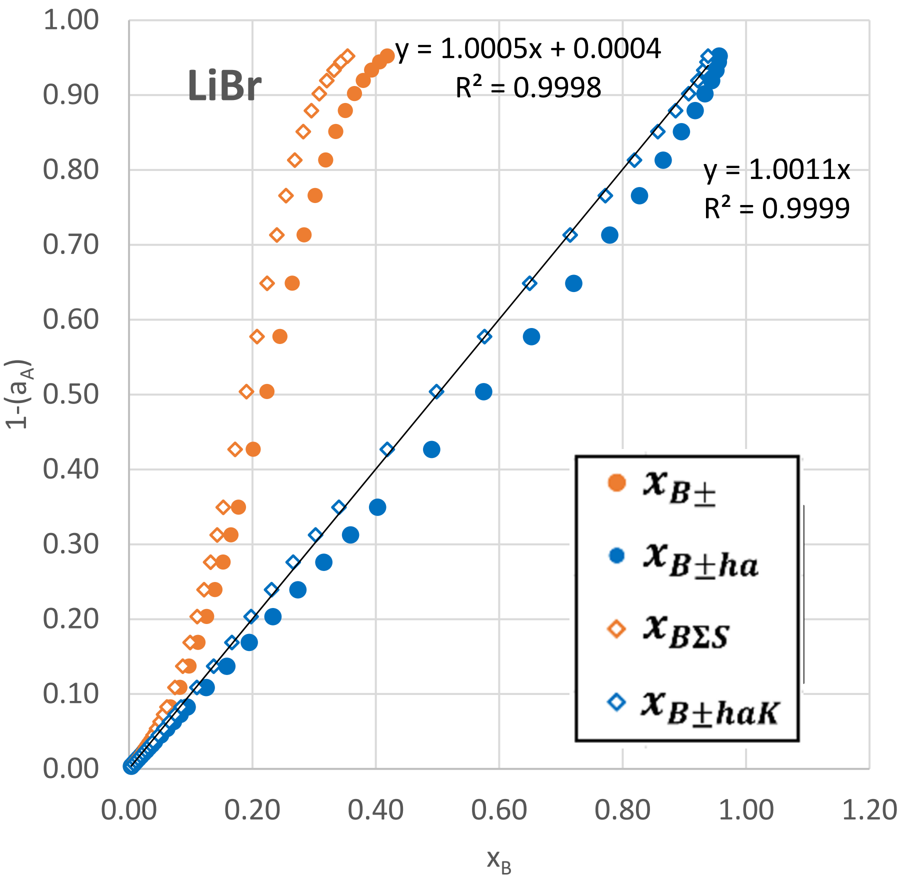

Figure S4. LiBr activity data (Robinson, R. A. & Stokes, R. H. *Electrolyte Solutions: Second Revised Edition*. (Dover Publications, Incorporated, 2012)) fit to $K_{id} = 0.12(\text{fixed})$, $K_{ha} = 5.08$, *m* =2.45.

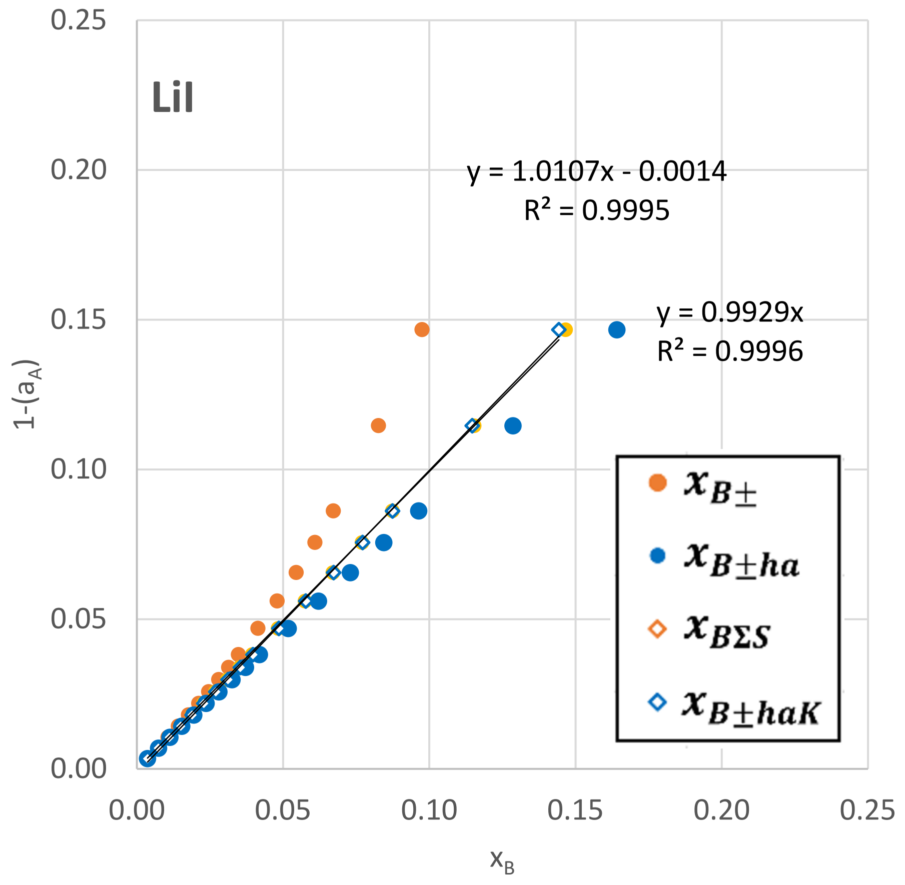

Figure S5. LiI activity data (Robinson, R. A. & Stokes, R. H. *Electrolyte Solutions: Second Revised Edition*. (Dover Publications, Incorporated, 2012)) fit to $K_{id} = 0.15\text{(fixed)}$, $K_{ha} = 5.32$, *m* =2.40.

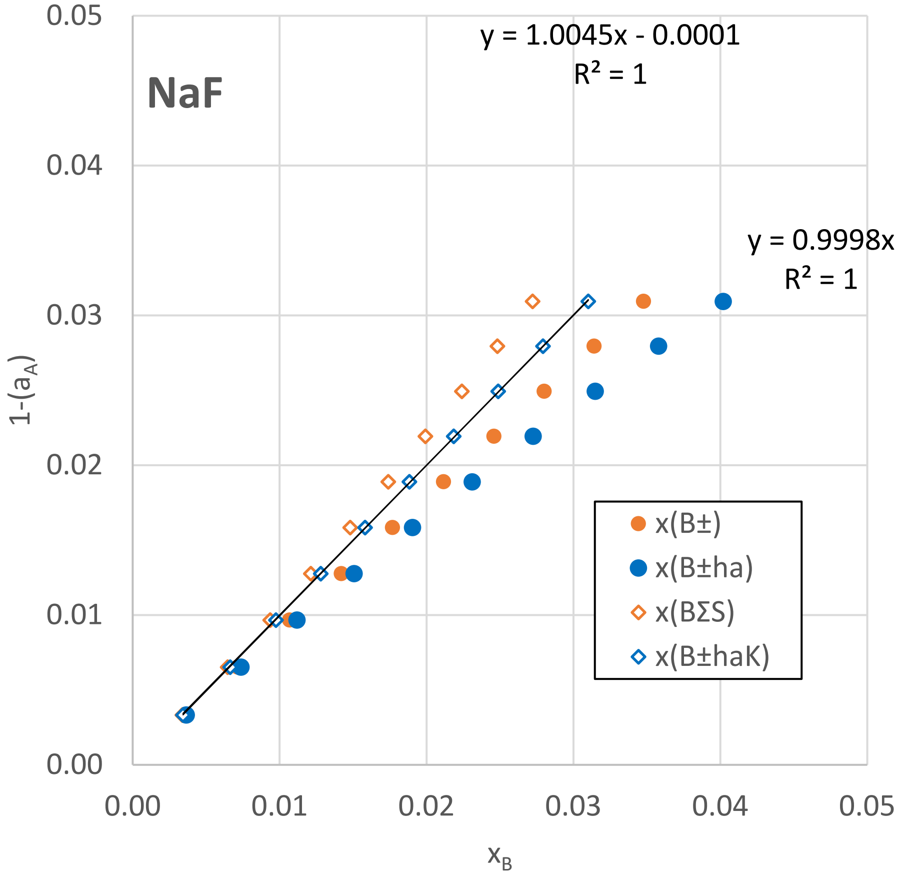

Figure S6. NaF activity data (Robinson, R. A. & Stokes, R. H. *Electrolyte Solutions: Second Revised Edition*. (Dover Publications, Incorporated, 2012)) fit to $K_{id} = 0.012$, $K_{ha} = 4.16$.

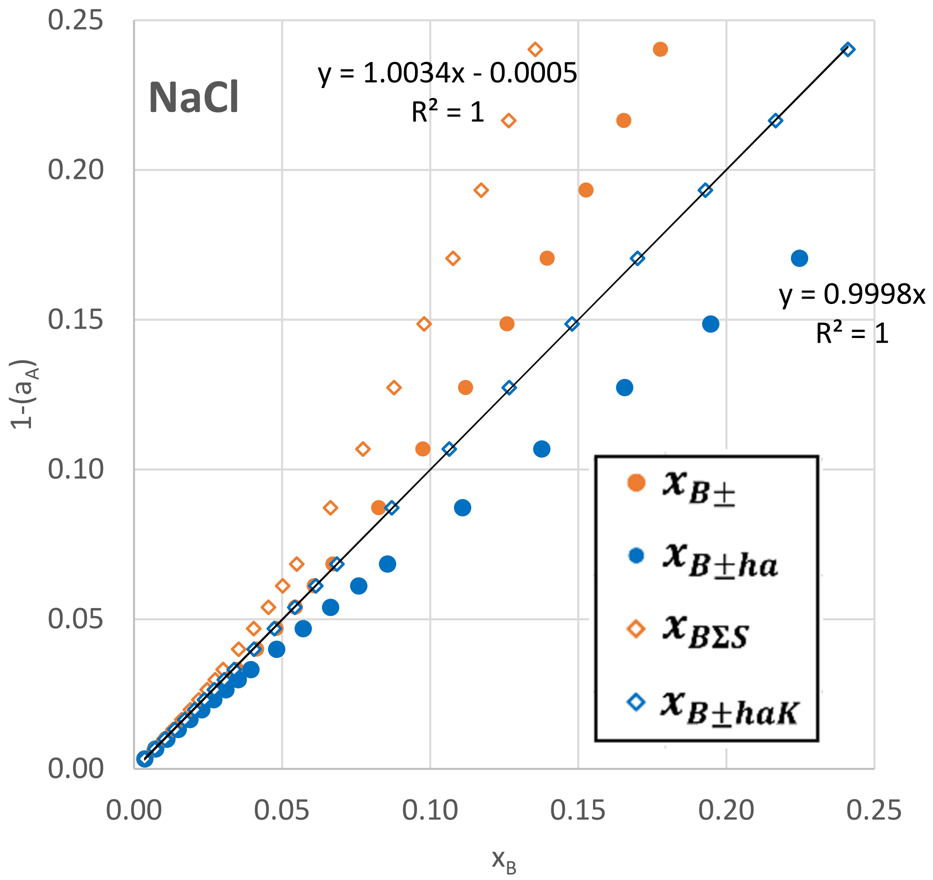

Figure S7. NaCl activity data (Robinson, R. A. & Stokes, R. H. *Electrolyte Solutions: Second Revised Edition*. (Dover Publications, Incorporated, 2012)) fit to $K_{id} = 0.0327$, $K_{ha} = 3.67$.

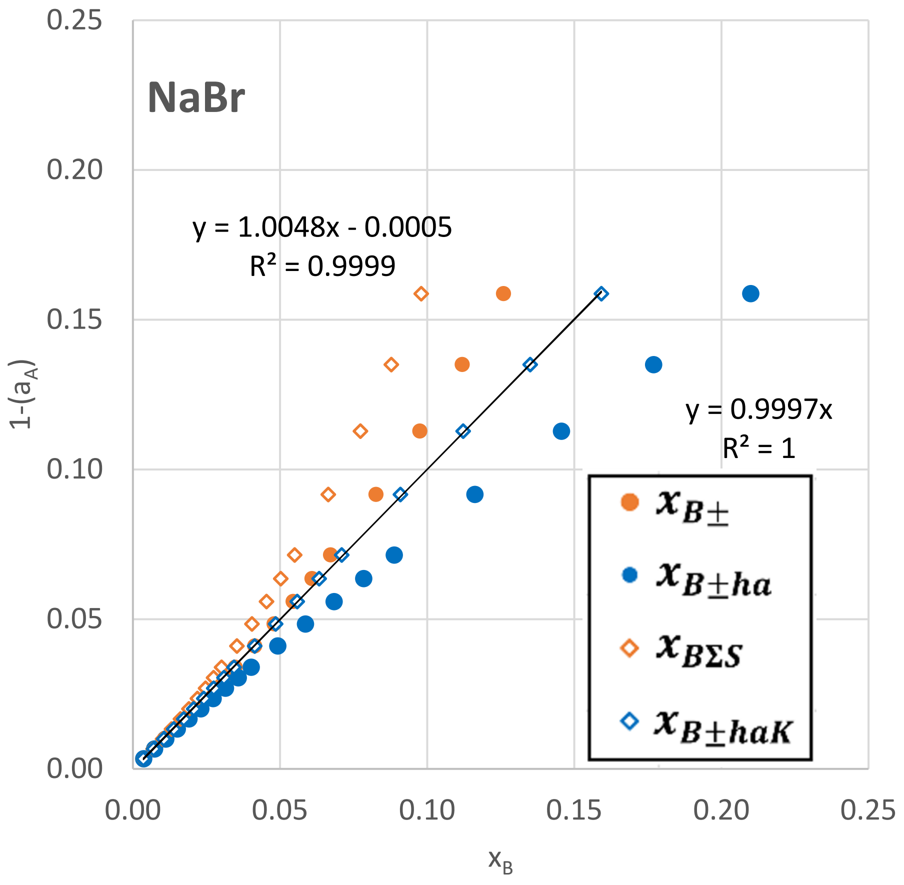

Figure S8. NaBr activity data (Robinson, R. A. & Stokes, R. H. *Electrolyte Solutions: Second Revised Edition*. (Dover Publications, Incorporated, 2012)) fit to $K_{id} = 0.0329$, $K_{ha} = 4.16$.

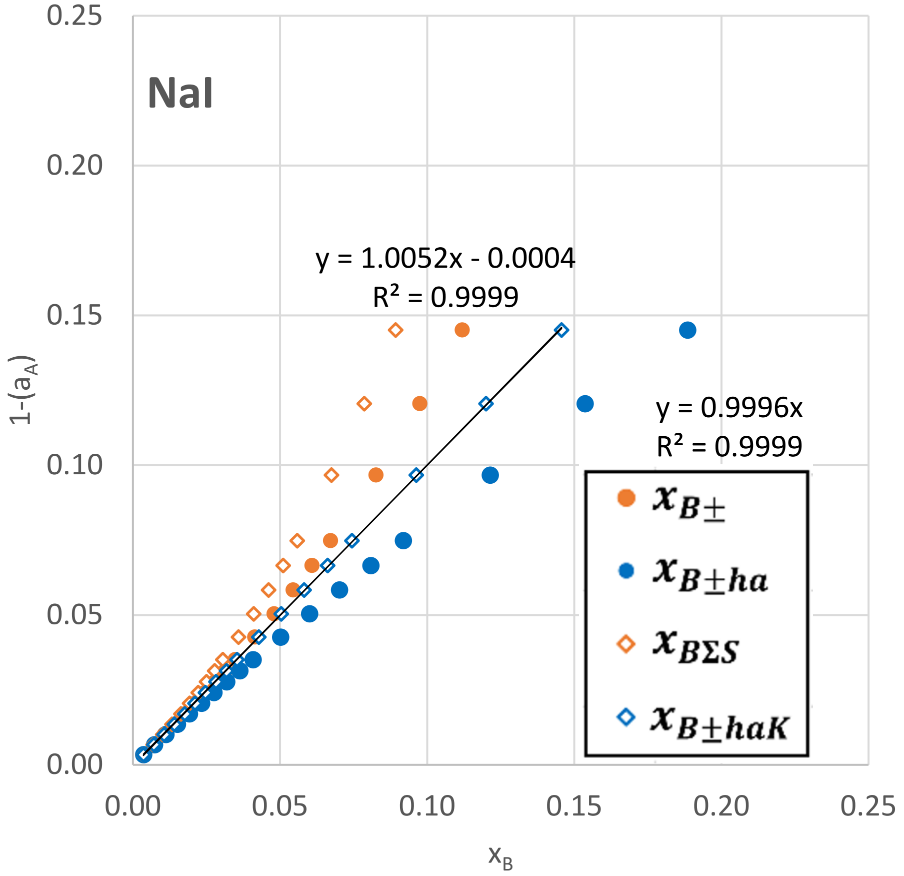

Figure S9. NaI activity data (Robinson, R. A. & Stokes, R. H. *Electrolyte Solutions: Second Revised Edition*. (Dover Publications, Incorporated, 2012)) fit to $K_{id} = 0.0388$, $K_{ha} = 4.60$.

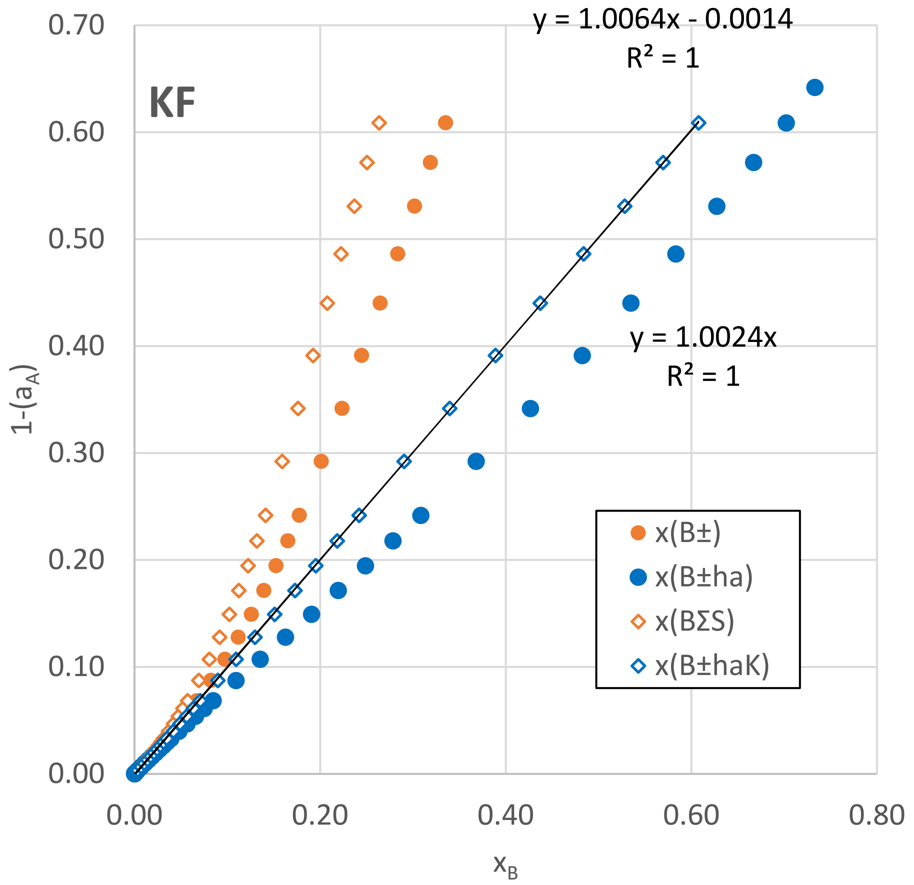

Figure S10. LiI activity data (W.J.W. Hamer, Osmotic coefficients and mean activity coefficients of a series of uni-univalent electrolytes in aqueous solutions at 25 deg C. Part 13 - Electrochemical data, 1969. https://ntrs.nasa.gov/search.jsp?R=19690029307) fit to $K_{id} = 0.051$, $K_{ha} = 3.53$.

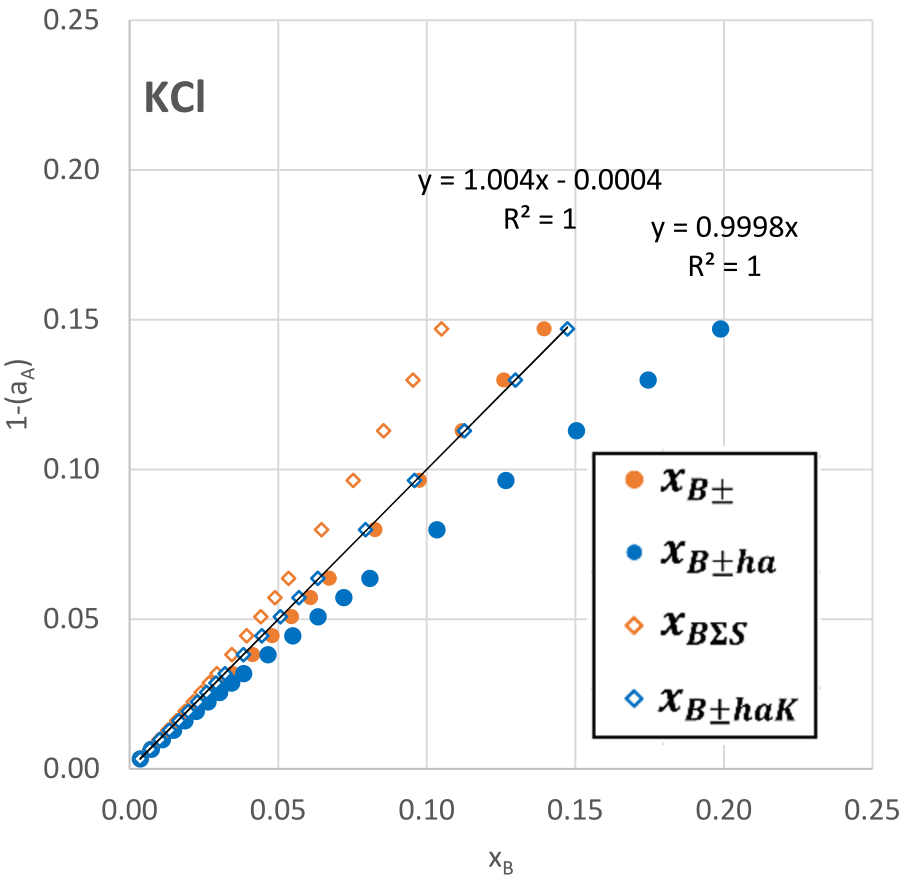

Figure S11. KCl activity data (Robinson, R. A. & Stokes, R. H. *Electrolyte Solutions: Second Revised Edition*. (Dover Publications, Incorporated, 2012)) fit to $K_{id} = 0.0252$, $K_{ha} = 2.89$.

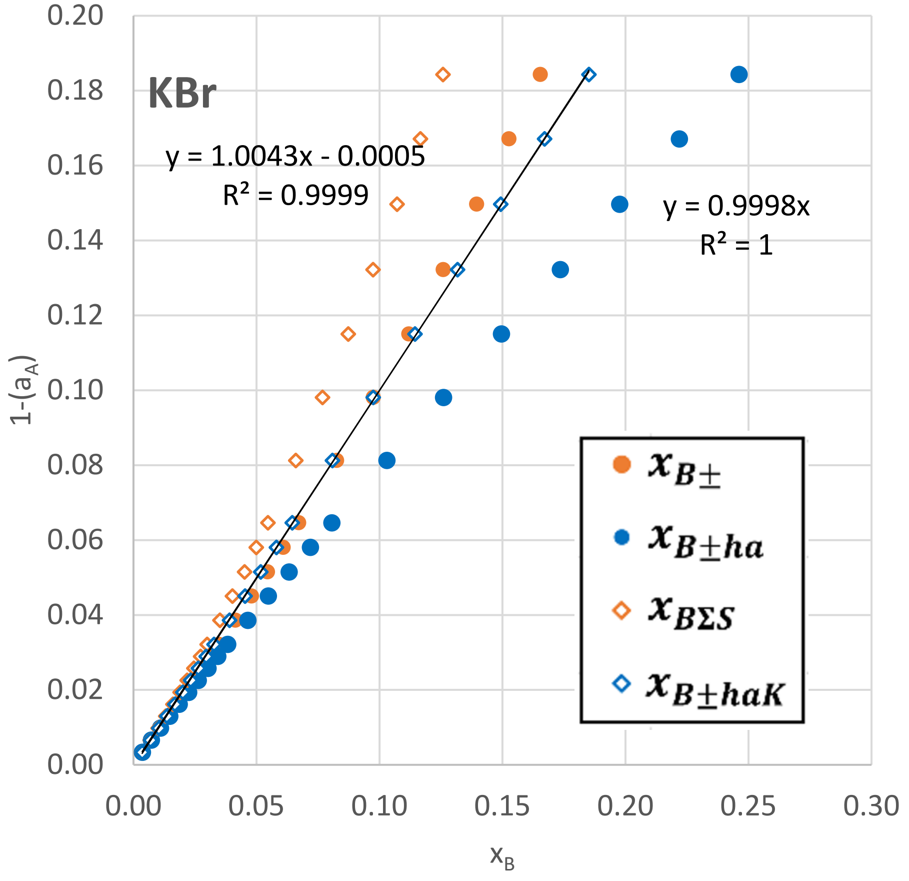

Figure S12. KBr activity data (Robinson, R. A. & Stokes, R. H. *Electrolyte Solutions: Second Revised Edition*. (Dover Publications, Incorporated, 2012)) fit to $K_{id} = 0.031$, $K_{ha} = 2.85$.

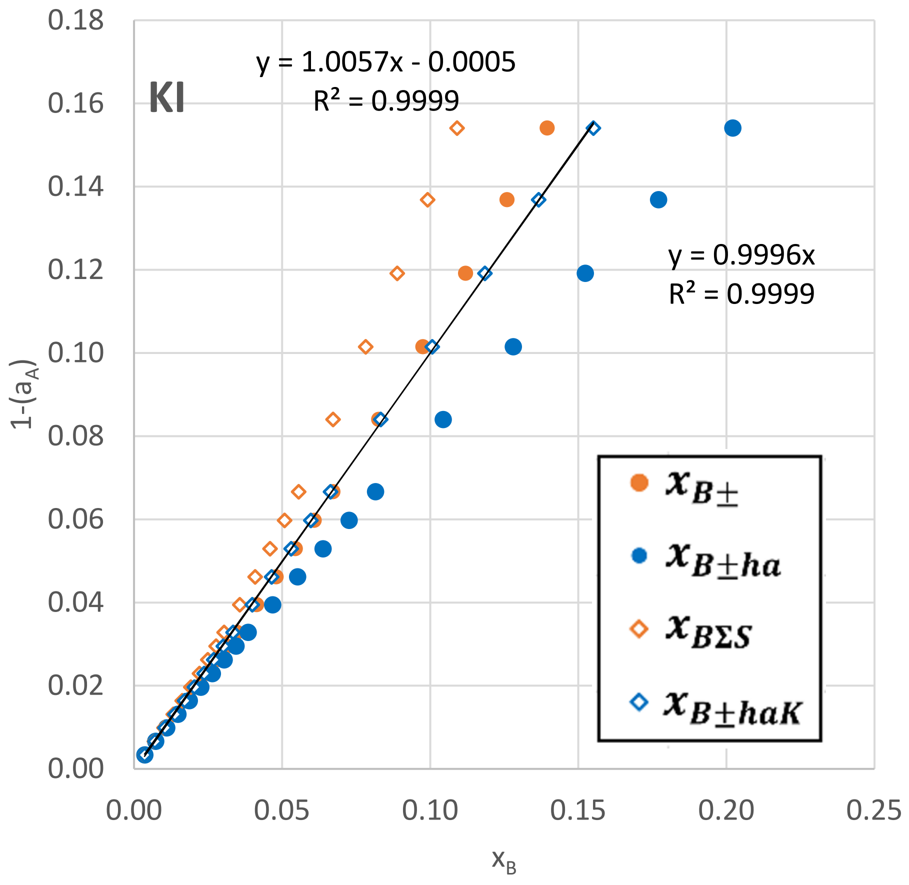

Figure S13. KI activity data (Robinson, R. A. & Stokes, R. H. *Electrolyte Solutions: Second Revised Edition*. (Dover Publications, Incorporated, 2012)) fit to $K_{id} = 0.0370$, $K_{ha} = 3.00$.

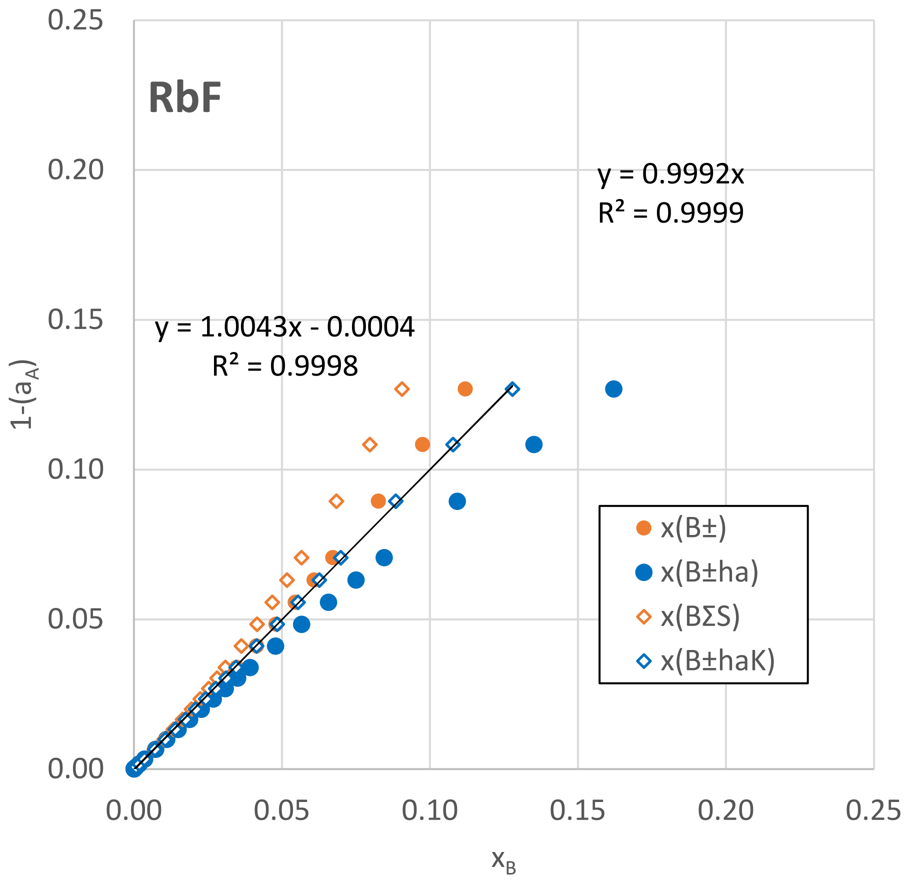

Figure S14. RbF activity data (H.T. Tien, THE ACTIVITY COEFFICIENTS OF RUBIDIUM AND CESIUM FLUORIDES IN AQUEOUS SOLUTION FROM VAPOR PRESSURE MEASUREMENTS, J. Phys. Chem. 67 (1963) 532–533. https://doi.org/10.1021/j100796a527) fit to $K_{id} = 0.045$, $K_{ha} = 3.50$.

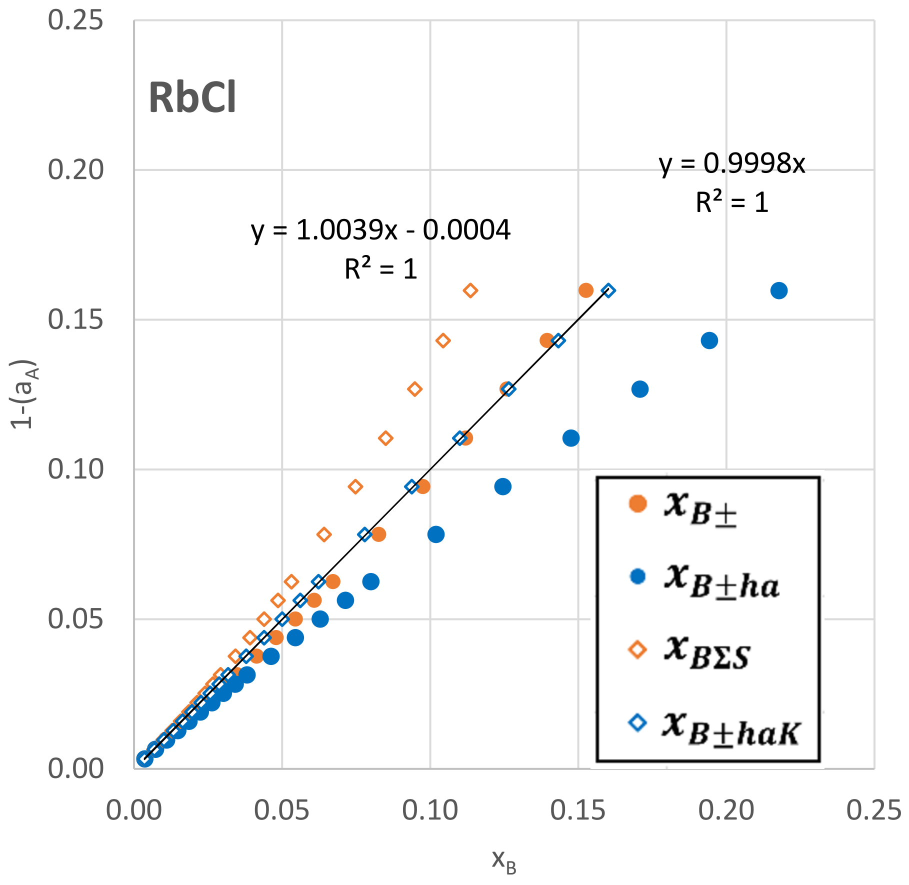

Figure S15. RbCl activity data (Robinson, R. A. & Stokes, R. H. *Electrolyte Solutions: Second Revised Edition*. (Dover Publications, Incorporated, 2012)) fit to $K_{id} = 0.0172$, $K_{ha} = 2.06$.

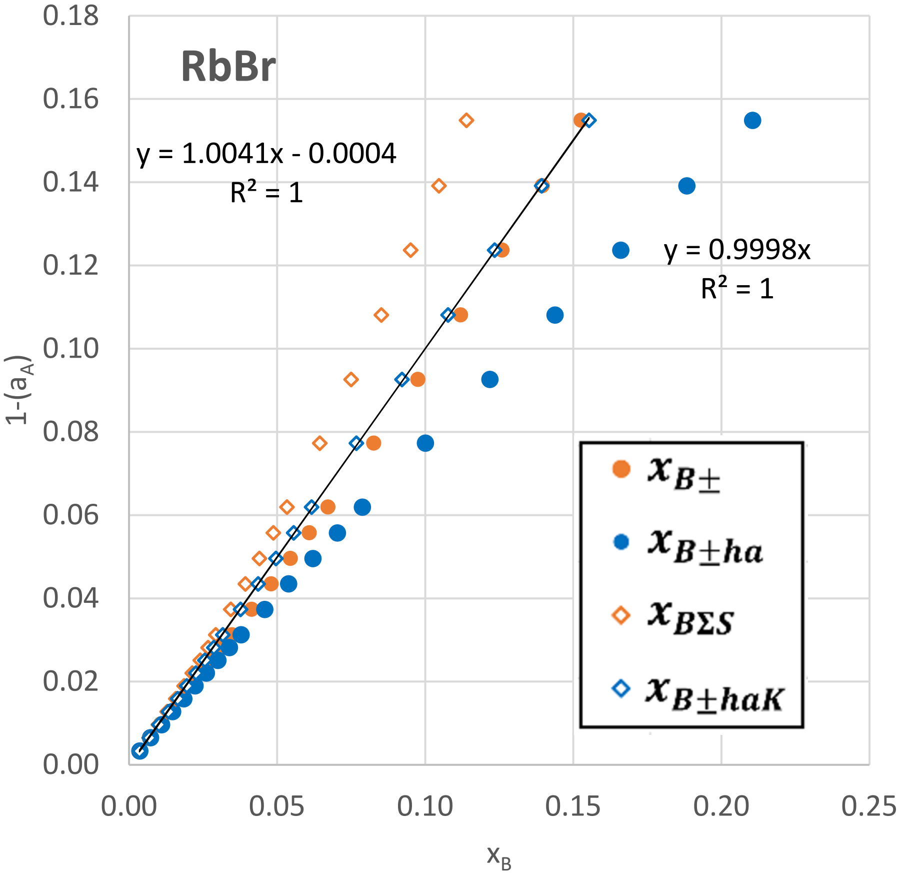

Figure S16. RbBr activity data (Robinson, R. A. & Stokes, R. H. *Electrolyte Solutions: Second Revised Edition*. (Dover Publications, Incorporated, 2012)) fit to $K_{id} = 0.0244$, $K_{ha} = 2.51$.

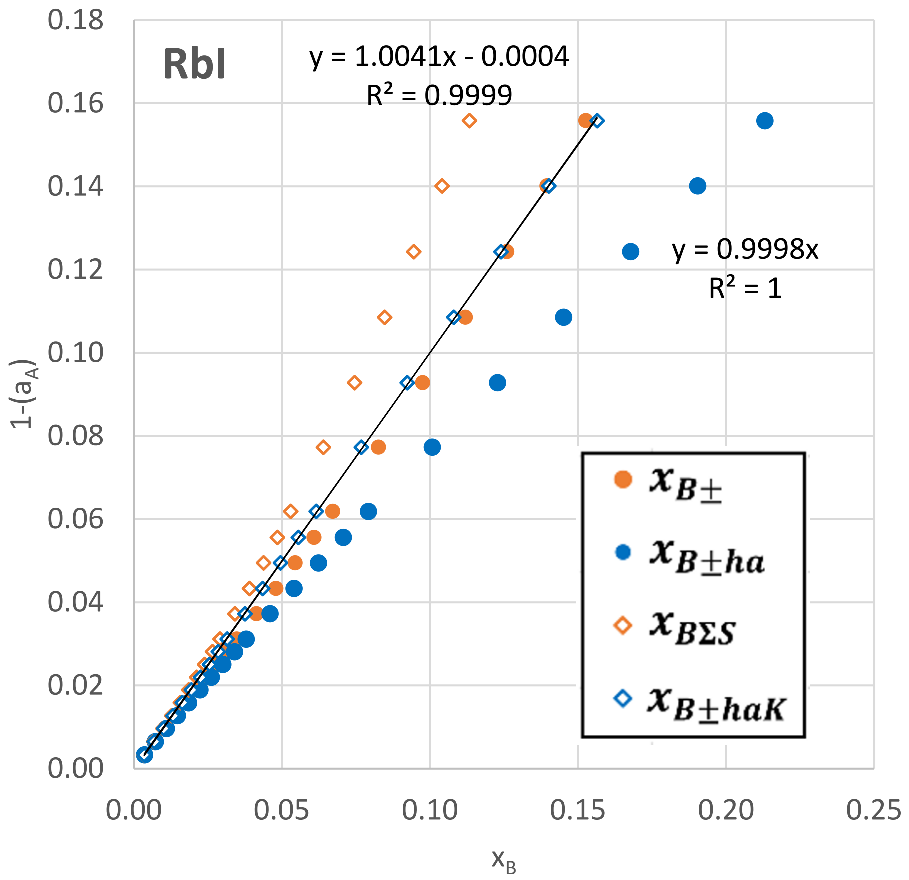

Figure S17. RbI activity data (Robinson, R. A. & Stokes, R. H. *Electrolyte Solutions: Second Revised Edition*. (Dover Publications, Incorporated, 2012)) fit to $K_{id} = 0.0231$, $K_{ha} = 2.59$.

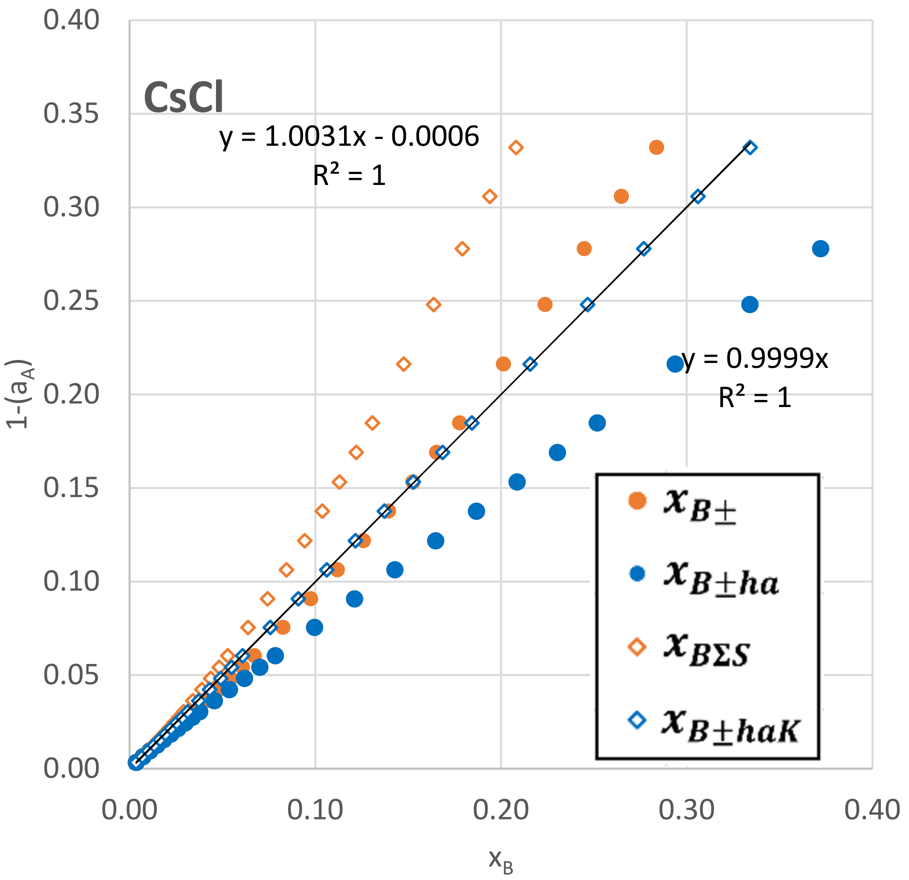

Figure S18. CsCl activity data (Robinson, R. A. & Stokes, R. H. *Electrolyte Solutions: Second Revised Edition*. (Dover Publications, Incorporated, 2012)) fit to $K_{id} = 0.0224$, $K_{ha} = 2.45$.

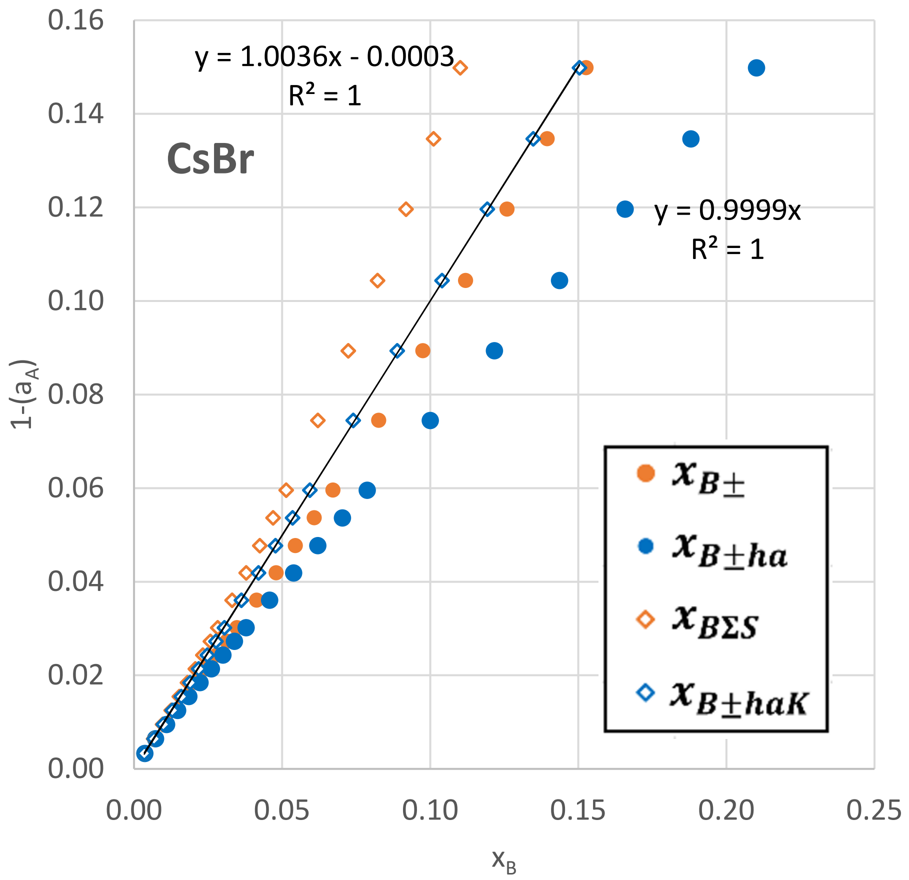

Figure S19. CsBr activity data (Robinson, R. A. & Stokes, R. H. *Electrolyte Solutions: Second Revised Edition*. (Dover Publications, Incorporated, 2012)) fit to $K_{id} = 0.0171$, $K_{ha} = 2.50$.

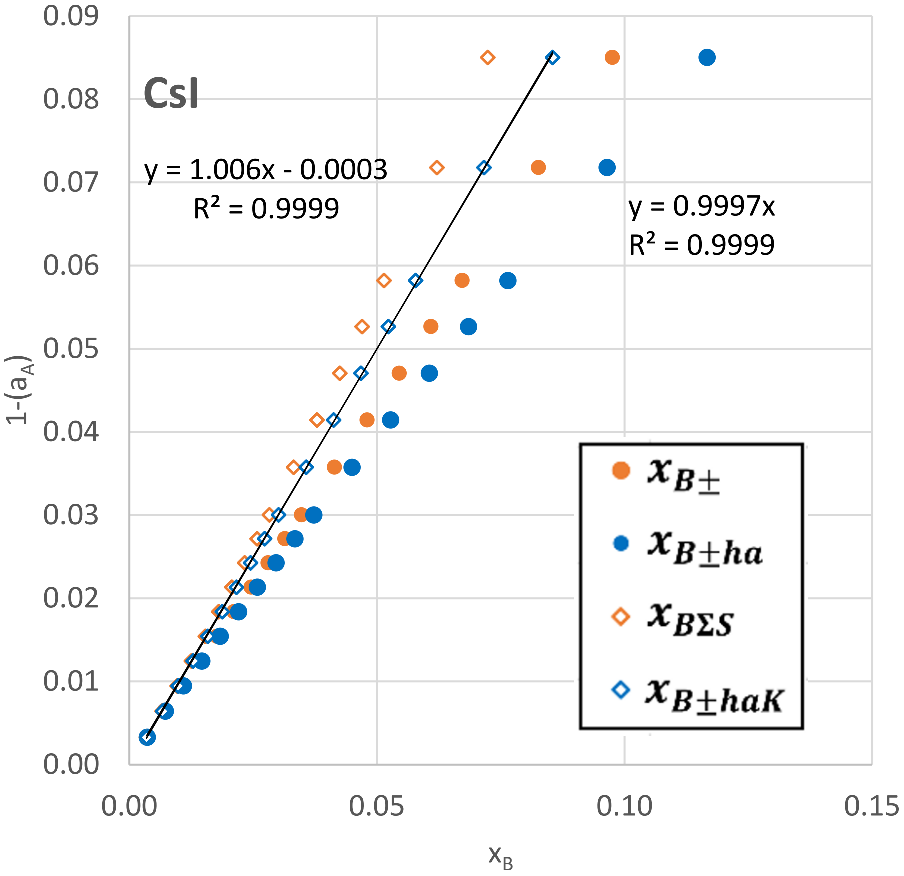

Figure S20. CsI activity data (Robinson, R. A. & Stokes, R. H. *Electrolyte Solutions: Second Revised Edition*. (Dover Publications, Incorporated, 2012)) fit to $K_{id} = 0.0172$, $K_{ha} = 2.06$.

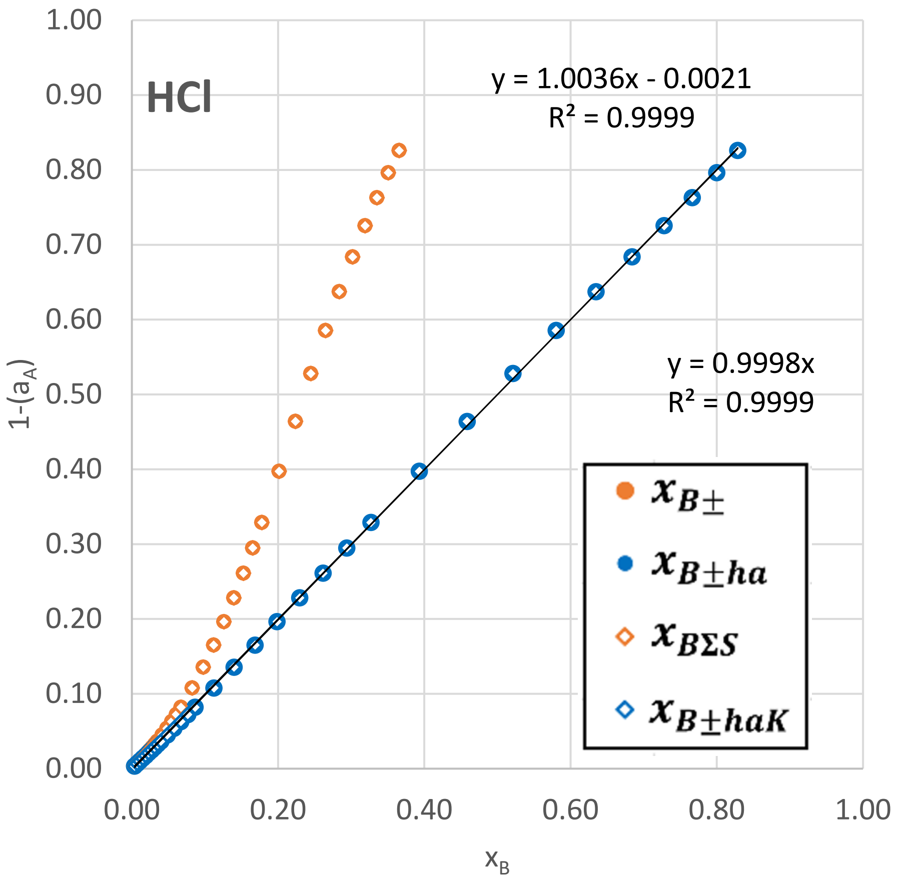

Figure S21. HCl activity data (Robinson, R. A. & Stokes, R. H. *Electrolyte Solutions: Second Revised Edition*. (Dover Publications, Incorporated, 2012)) fit to $K_{id} = 10^{6.3}$ (fixed), $K_{ha} = 3.80$.

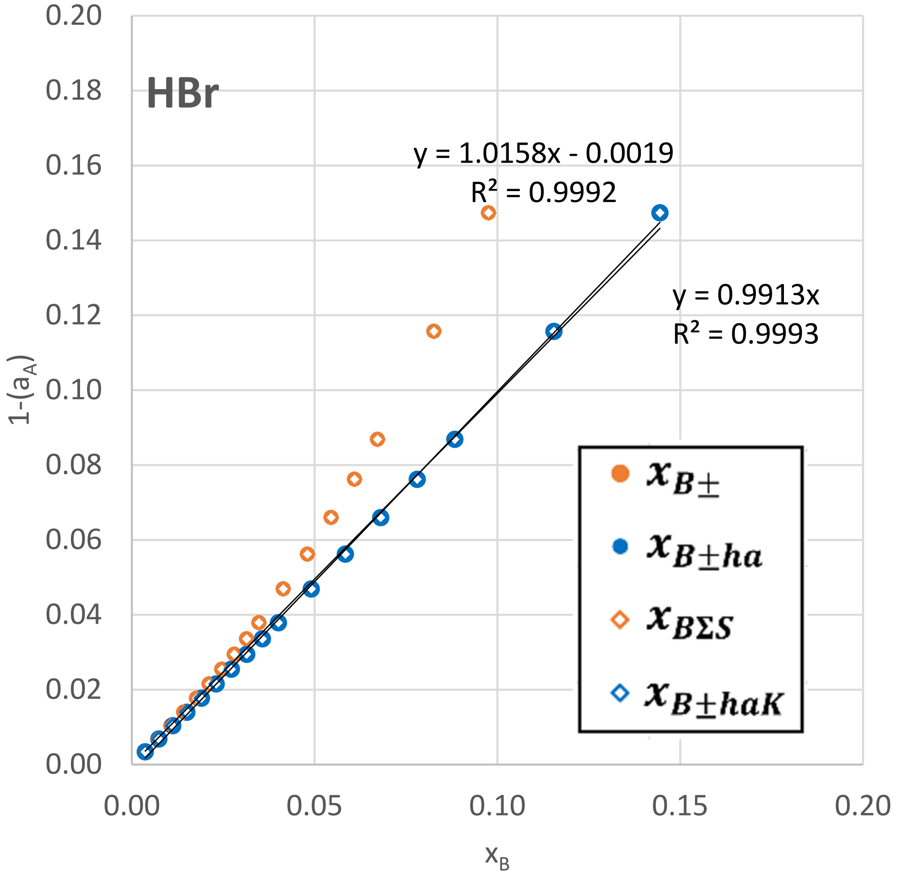

Figure S22. HBr activity data (Robinson, R. A. & Stokes, R. H. *Electrolyte Solutions: Second Revised Edition*. (Dover Publications, Incorporated, 2012)) fit to $K_{id} = 10^{8.7}$(fixed), $K_{ha} = 4.09$.

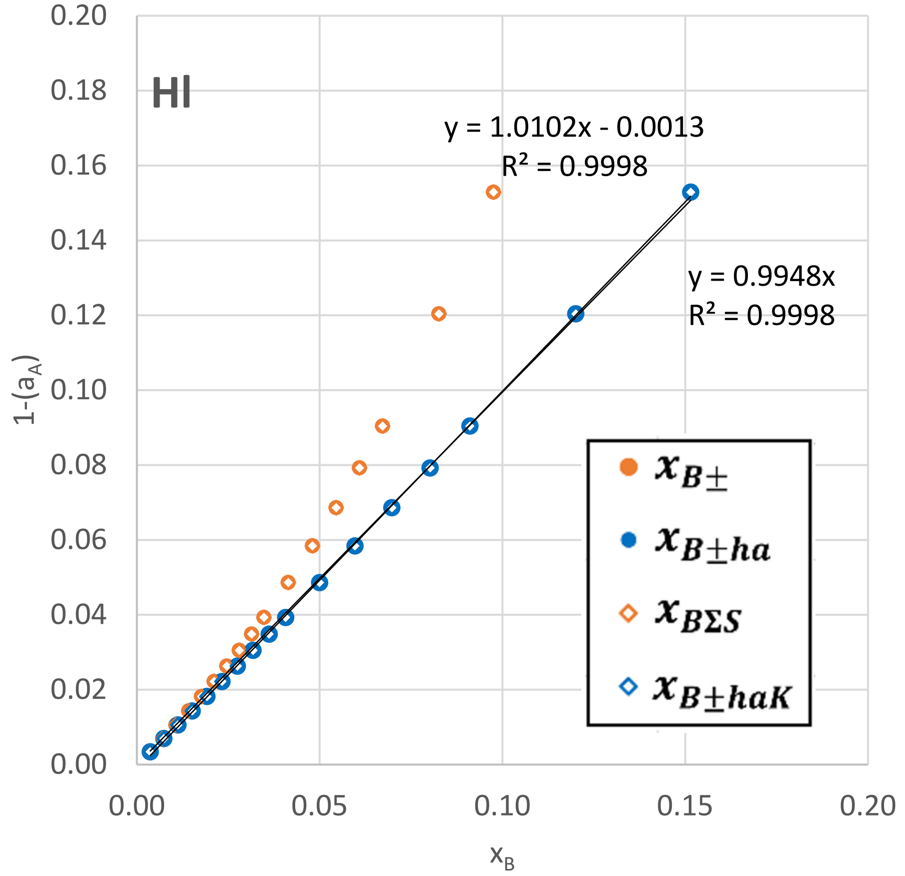

Figure S23. HI activity data (Robinson, R. A. & Stokes, R. H. *Electrolyte Solutions: Second Revised Edition*. (Dover Publications, Incorporated, 2012)) fit to $K_{id} = 10^{9.3}(\text{fixed})$, $K_{ha} = 4.48$.

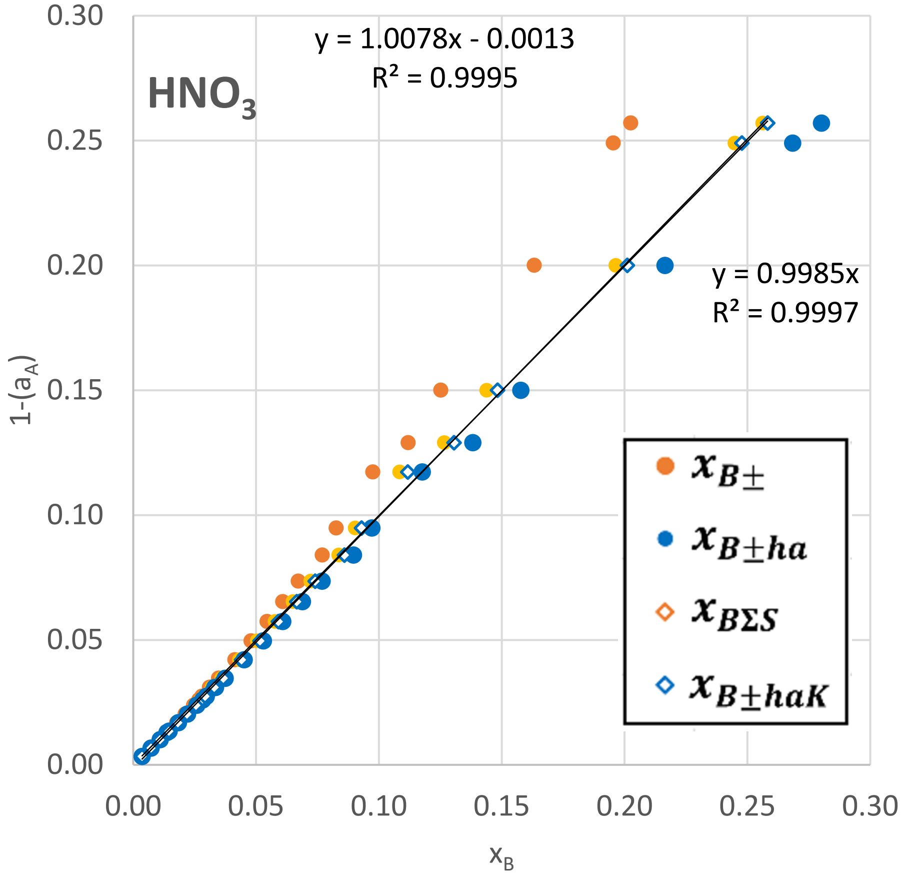

Figure S24. HNO_3 activity data (I.N. Tang, H.R. Munkelwitz, J.H. Lee, Vapor-liquid equilibrium measurements for dilute nitric acid solutions, Atmospheric Environment (1967). 22 (1988) 2579–2585. https://doi.org/10.1016/0004-6981(88)90491-X.) fit to $K_{id} = 0.414$, $K_{ha} = 2.15$.

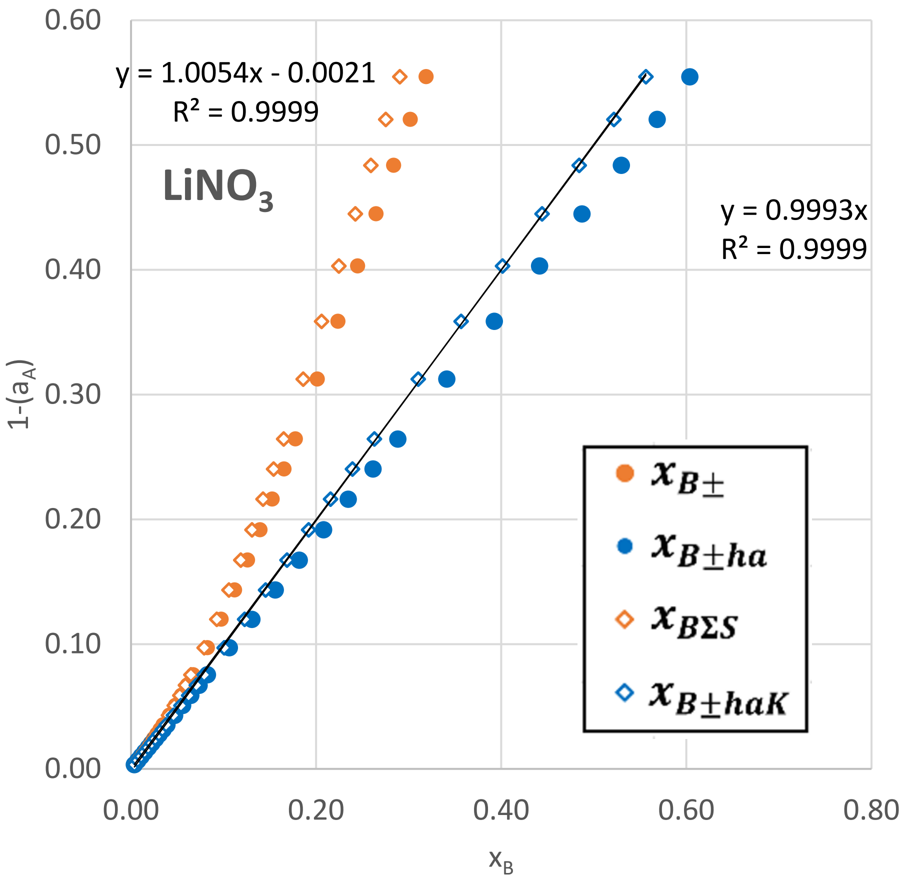

Figure S25. $LiNO_3$ activity data (Robinson, R. A. & Stokes, R. H. *Electrolyte Solutions: Second Revised Edition*. (Dover Publications, Incorporated, 2012)) fit to $K_{id} = 0.354$, $K_{ha} = 3.19$.

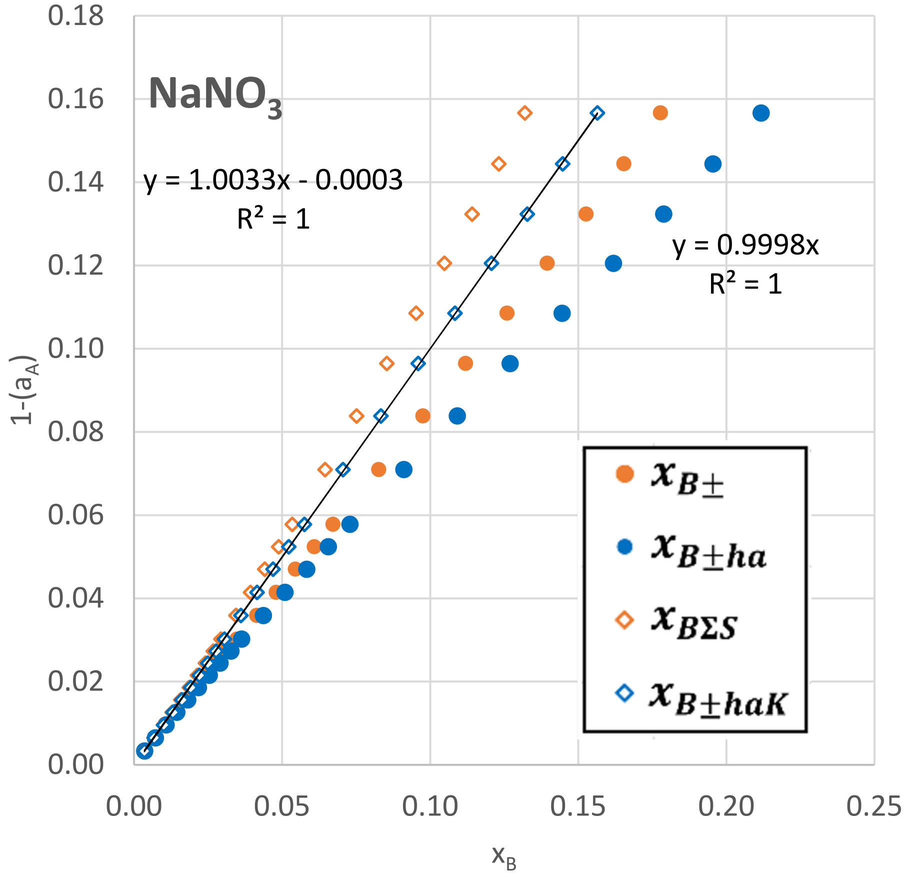

Figure S26. $NaNO_3$ activity data (Robinson, R. A. & Stokes, R. H. *Electrolyte Solutions: Second Revised Edition*. (Dover Publications, Incorporated, 2012)) fit to $K_{id} = 0.0248$, $K_{ha} = 1.34$.

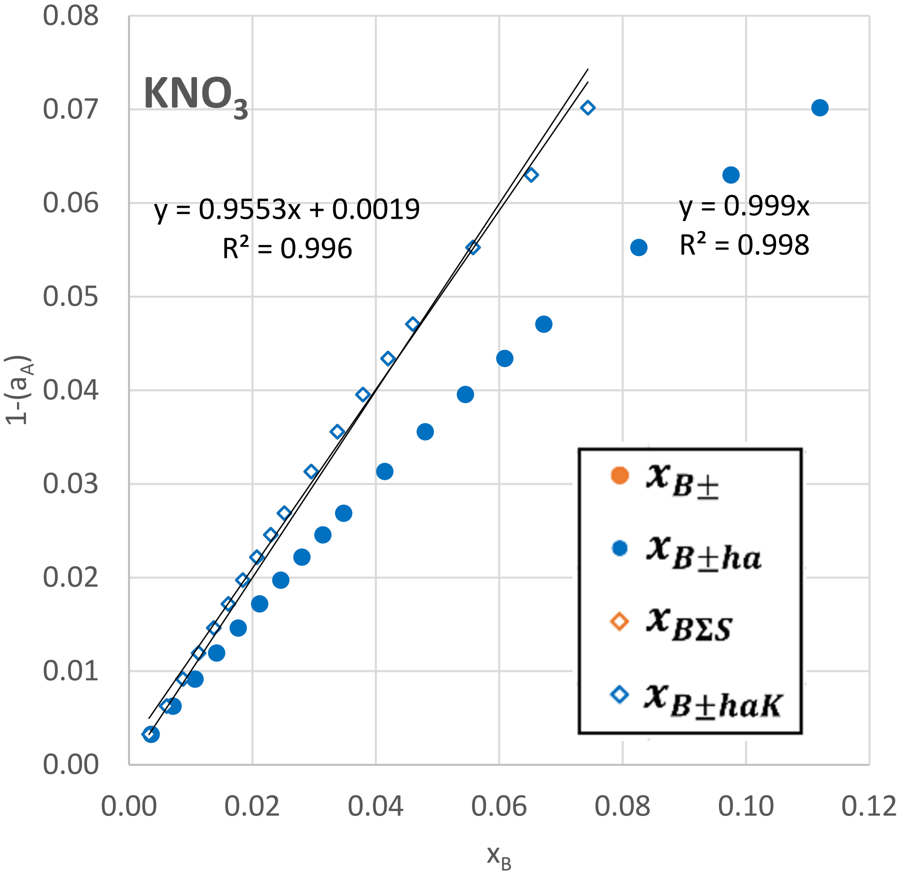

Figure S27. KNO_3 activity data (Robinson, R. A. & Stokes, R. H. *Electrolyte Solutions: Second Revised Edition*. (Dover Publications, Incorporated, 2012)) fit to $K_{id} = 0.0058$, $K_{ha} = 0.0$.

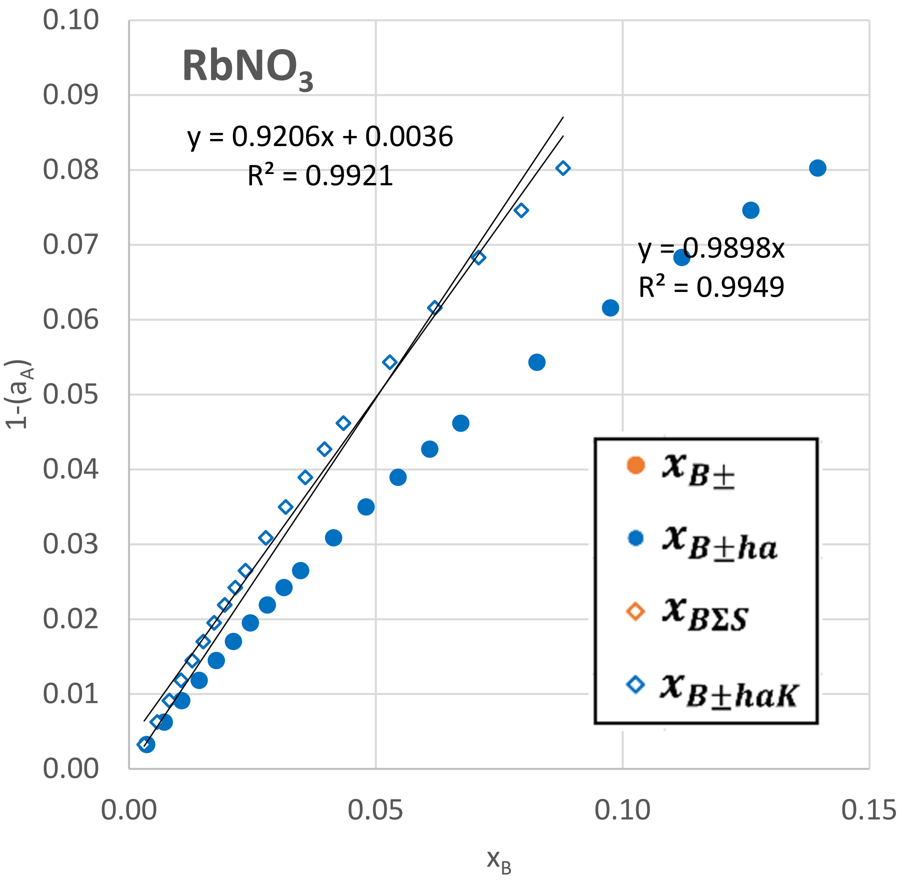

Figure S28. $RbNO_3$ activity data (Robinson, R. A. & Stokes, R. H. *Electrolyte Solutions: Second Revised Edition*. (Dover Publications, Incorporated, 2012)) fit to $K_{id} = 0.0031$, $K_{ha} = 0.0$.

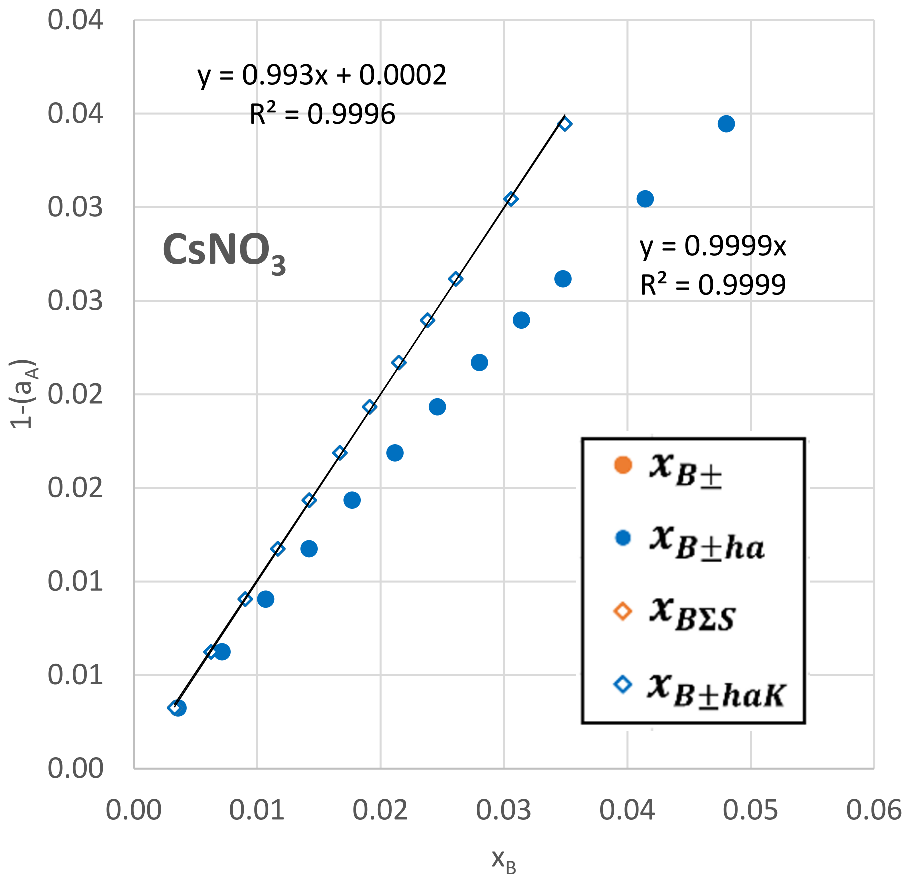

Figure S29. $CsNO_3$ activity data (Robinson, R. A. & Stokes, R. H. *Electrolyte Solutions: Second Revised Edition*. (Dover Publications, Incorporated, 2012)) fit to $K_{id} = 0.0080$, $K_{ha} = 0.0$.

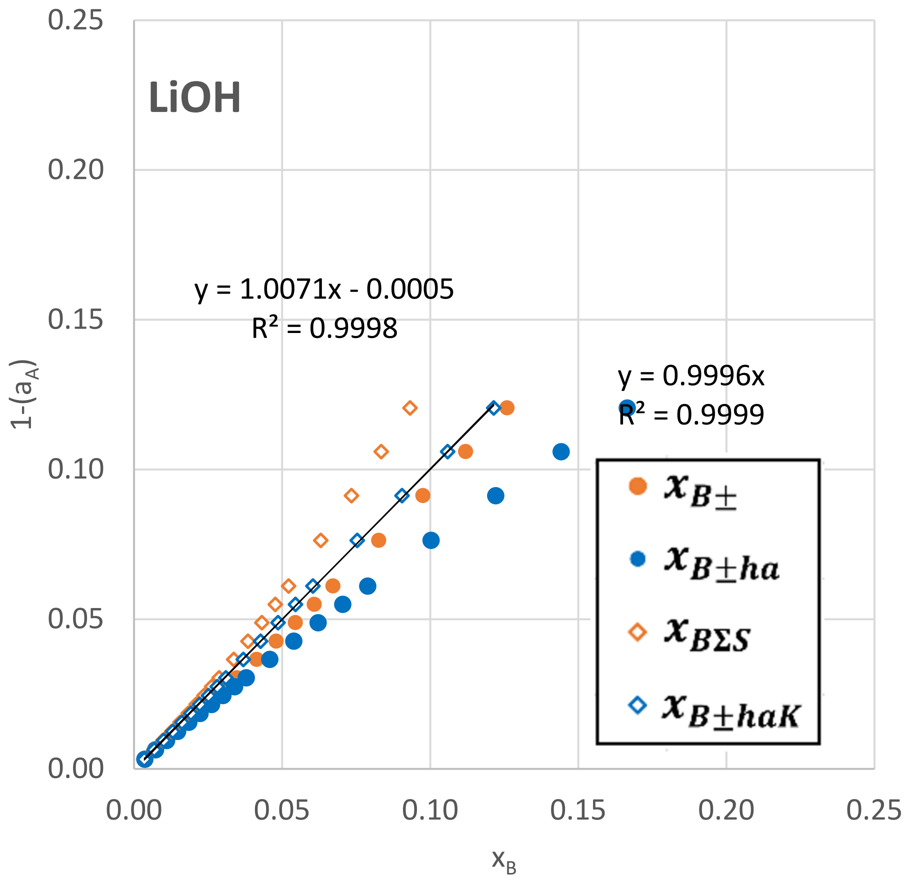

Figure S30. LiOH activity data (Robinson, R. A. & Stokes, R. H. *Electrolyte Solutions: Second Revised Edition*. (Dover Publications, Incorporated, 2012)) fit to $K_{id} = 0.0199$, $K_{ha} = 2.53$.

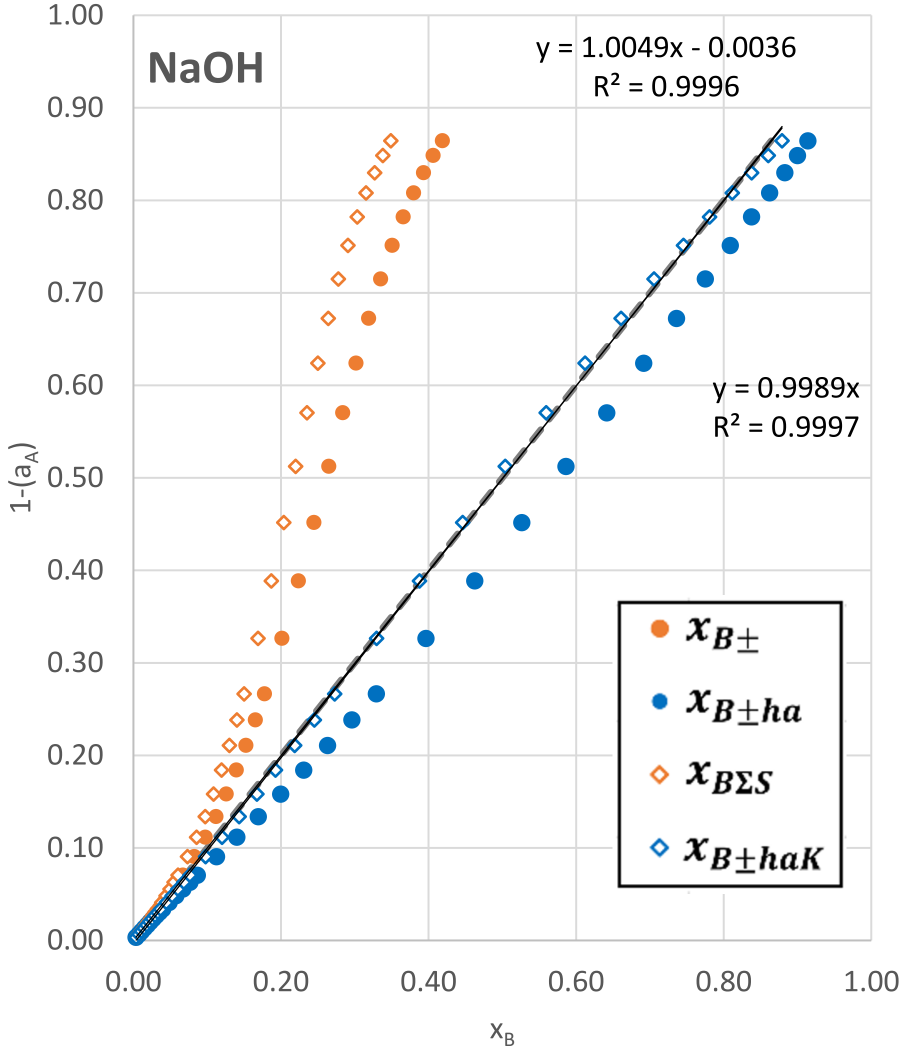

Figure S31. NaOH activity data (Robinson, R. A. & Stokes, R. H. *Electrolyte Solutions: Second Revised Edition*. (Dover Publications, Incorporated, 2012)) fit to $K_{id} = 0.097$, $K_{ha} = 3.83$.

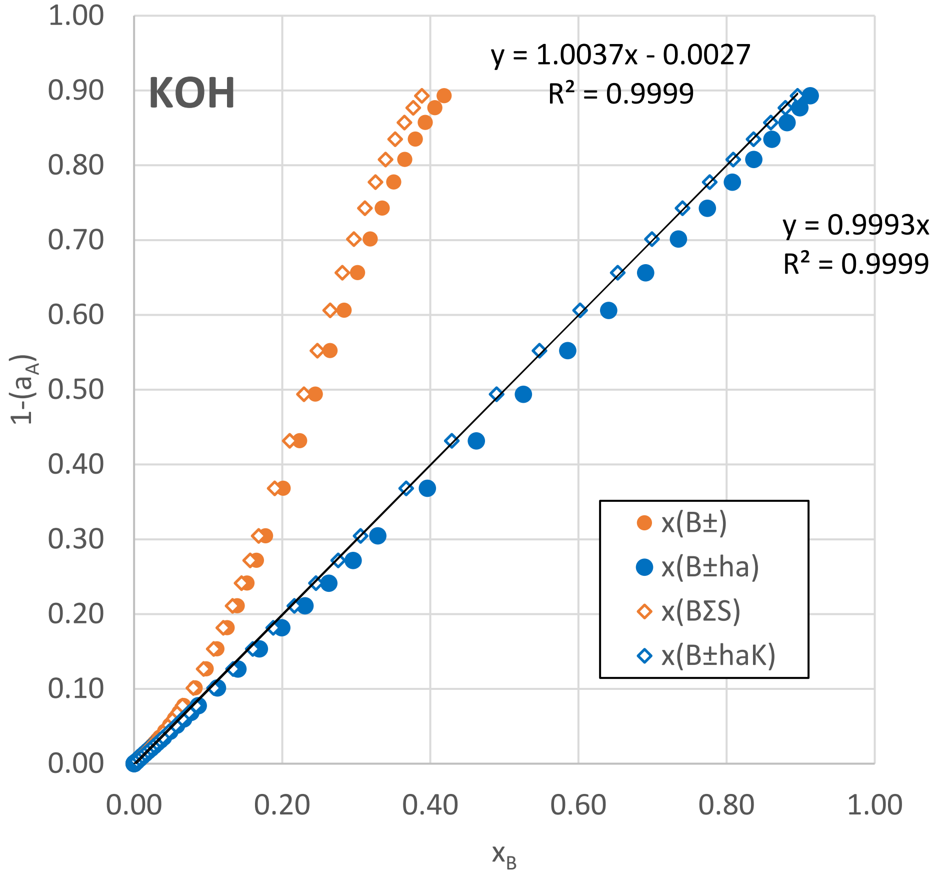

Figure S32. KOH activity data (W.J.W. Hamer, Osmotic coefficients and mean activity coefficients of a series of uni-univalent electrolytes in aqueous solutions at 25 deg C. Part 13 - Electrochemical data, 1969. https://ntrs.nasa.gov/search.jsp?R=19690029307) fit to $K_{id} = 0.52$, $K_{ha} = 3.82$.

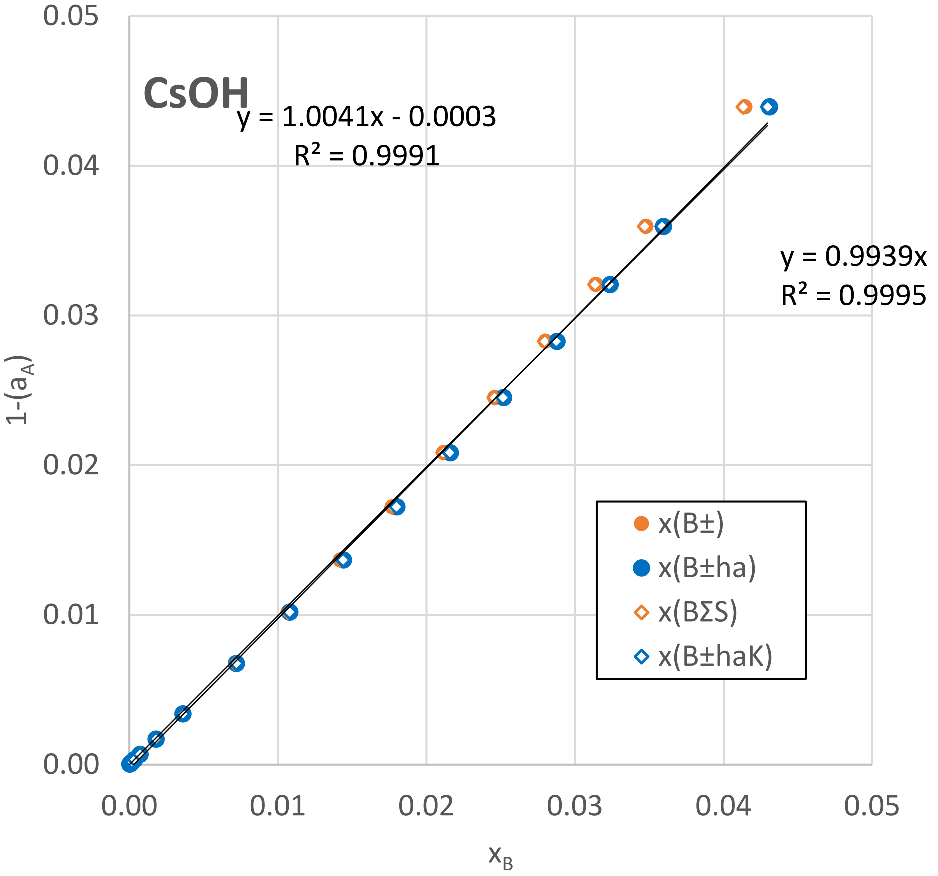

Figure S33. CsOH activity data (H.S. Harned, O.E. Schupp, THE ACTIVITY COEFFICIENTS OF CESIUM CHLORIDE AND HYDROXIDE IN AQUEOUS SOLUTION, J. Am. Chem. Soc. 52 (1930) 3886–3892. https://doi.org/10.1021/ja01373a019) fit to $K_{id} = 0.043$, $K_{ha} = 3.39$.

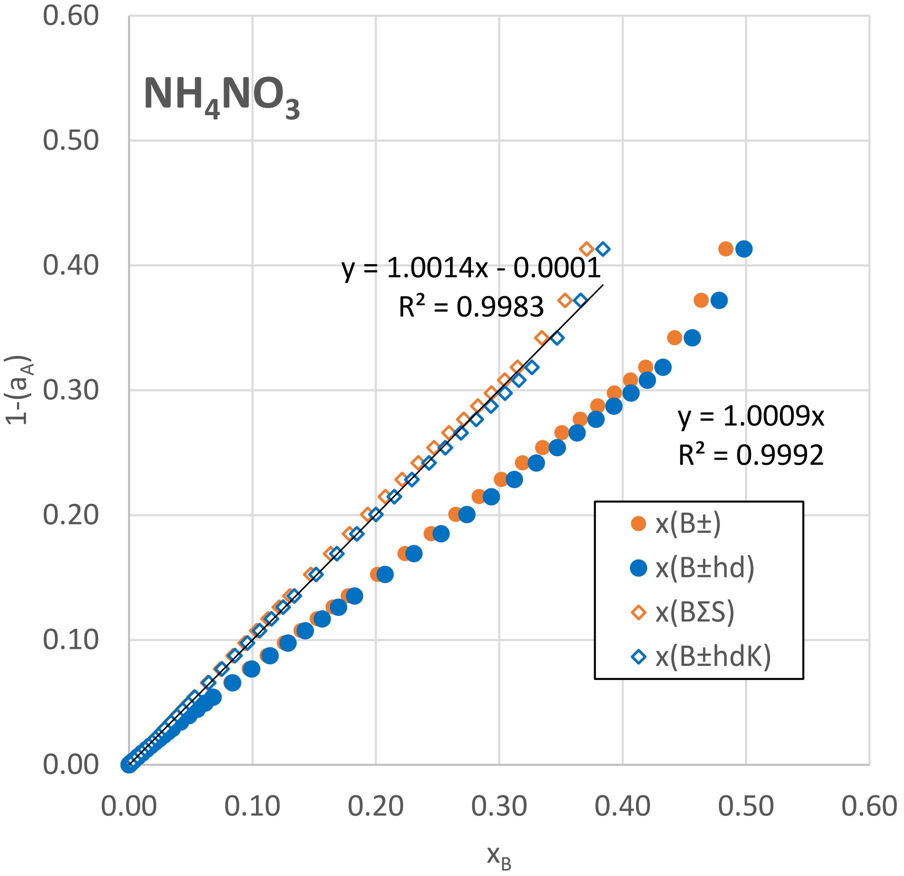

Figure S34. CsOH activity data (B.F. Wishaw, R.H. Stokes, The osmotic and activity coefficients of aqueous solutions of ammonium chloride and ammonium nitrate at 25°, Trans. Faraday Soc. 49 (1953) 27–31. https://doi.org/10.1039/TF9534900027) fit to $K_{id} = 0.022$, $K_{ha} = 0.23$.

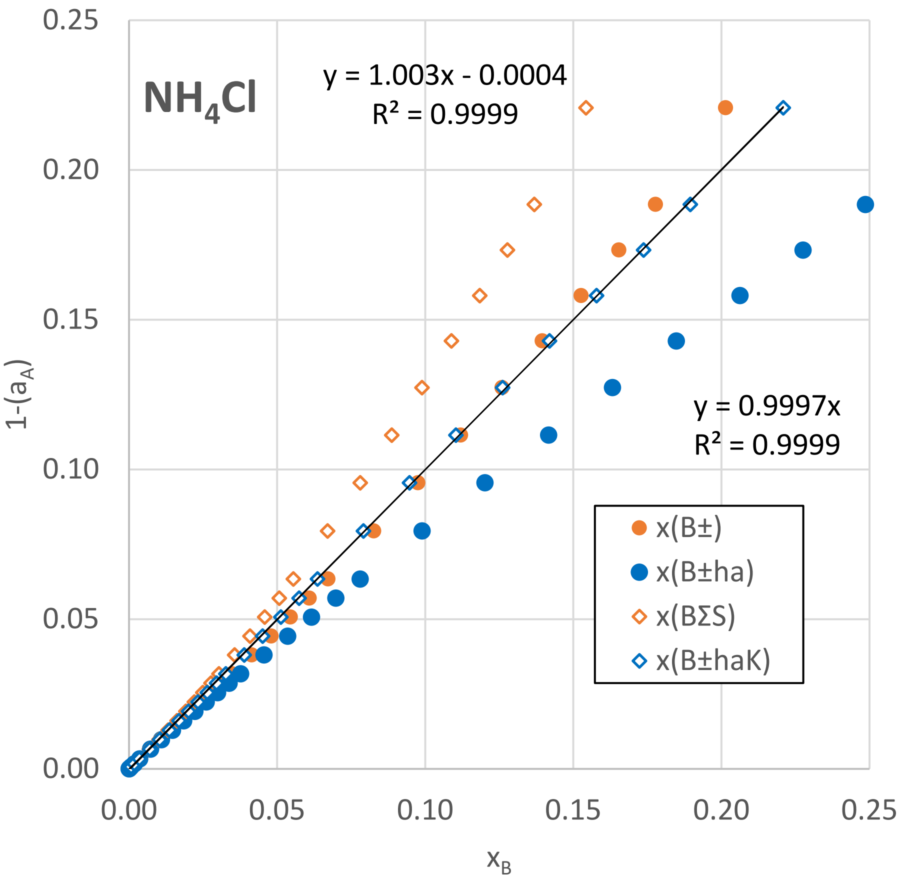

Figure S35. NH_4Cl activity data (B W.J.W. Hamer, Osmotic coefficients and mean activity coefficients of a series of uni-univalent electrolytes in aqueous solutions at 25 deg C. Part 13 - Electrochemical data, 1969. https://ntrs.nasa.gov/search.jsp?R=19690029307) fit to $K_{id} = 0.036$, $K_{ha} = 2.37$.

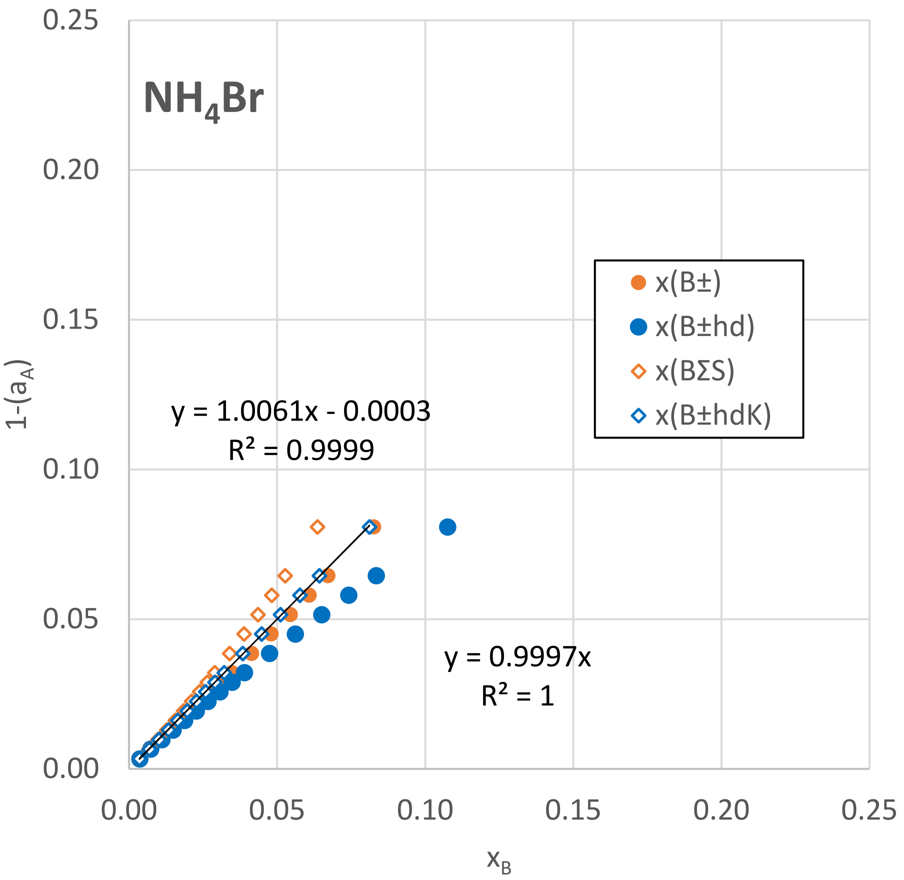

Figure S36. NH_4Br activity data (A.K. Covington, D.E. Irish, Osmotic and activity coefficients of aqueous ammonium bromide solutions at 25.deg., J. Chem. Eng. Data. 17 (1972) 175–176. https://doi.org/10.1021/je60053a034) fit to $K_{id} = 0.022$, $K_{ha} = 3.34$.
